# A visit to The Doctor

**DOI:** 10.15694/mep.2018.0000191.1

**Published:** 2018-09-04

**Authors:** George Zaharias

**Affiliations:** 1 Royal Australian College of General Practitioners

**Keywords:** the arts, humanities, medical education, transformational learning, transformational change

## Abstract

This article was migrated. The article was marked as recommended.

Whether the arts have any value in the medical curriculum continues to be debated. This article presents the author’s views on the use of the arts, the principal contention being that the arts (the humanities) can teach us a lot about the art of medicine. In this article, Thomas Fildes’ painting, *The Doctor,* and Randa Haines’ film of the same name are referenced to demonstrate how the arts may be utilized in order to create broader awareness and understanding and ultimately, transformational learning and change, two quite powerful outcomes. How the arts should be incorporated into the medical curriculum and how they should be taught are definite issues. This article makes recommendations with respect to some of the difficulties associated with both.

## Thomas Fildes’ The Doctor

Luke Fildes, *The Doctor* (1891)

[The image is in the public domain, accessed August 2018, from: https://en.wikipedia.org/wiki/The_Doctor_(painting)]

**Figure:**
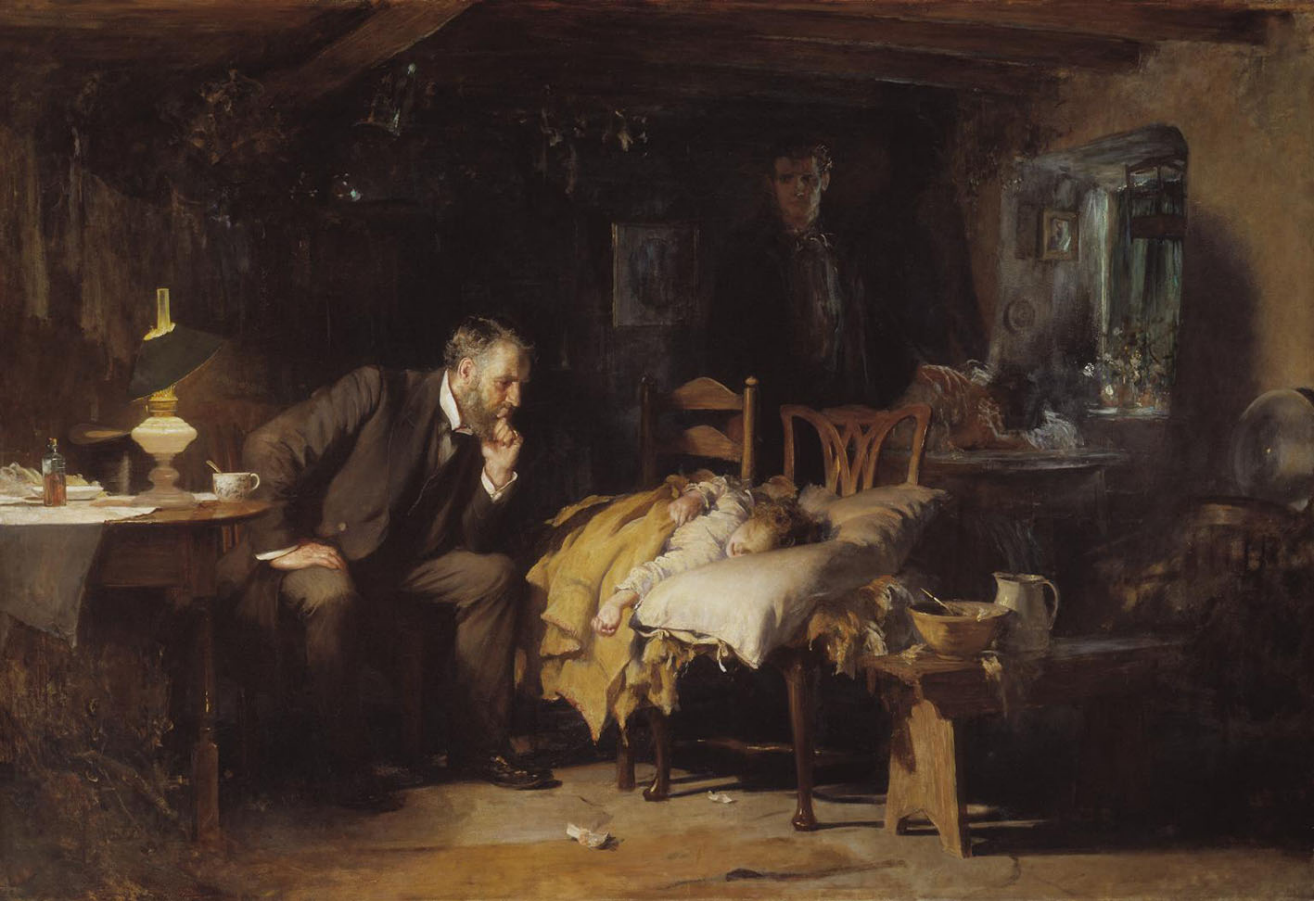


## Introduction

Thomas Fildes’ *The Doctor* is an iconic painting which portrays many of the qualities of a good doctor: calm, patient, caring, concerned, altruistic. It has appeared, unsurprisingly, on the cover of certain medical texts ( Moore AR, 1978; Montgomery, 2006) as well as an issue of *Academic Medicine* (June 2002) which had a focus on professionalism. It has been the subject of two journal articles ( Moore J, 2008; Barrett, 2010) which discussed how realistic or romanticized the depiction of the doctor is and which pointed out that it, “..reminds contemporary doctors of the crucial importance of the relationship between a patient and the doctor..” ( Moore J, 2008) and “..it still has a great deal to say about the vocational nature of medicine”. ( Barrett, 2010)

Barrett however, laments that, “..the high water mark of public perception represented by *The Doctor* has long passed.” ( Barrett, 2010) In the film, *The Doctor,* ( Haines, 1991) Dr Jack MacKee, the central character, gives credence to Barrett’s lament. He behaves exceedingly unprofessionally towards his patients and towards certain colleagues. His world is suddenly upturned however when he is diagnosed with cancer. He strikes an unlikely friendship with a young woman who also has cancer. He empathises with her and reflects on his own predicament. Subsequently *,* there is a scene where Dr MacKee, approaches the surgeon to whom he has openly behaved disrespectfully and asks him to operate on him. Despite a touch of black humor in the verbal exchange, it is a very telling scene as regards the sick role and professional behaviour.

Dr MacKee’s behaviour prior to his transformation raises many questions. Why did he have to go through this illness experience in order to discover humility and become a caring doctor? Why had he not learnt anything earlier from his patients’ sufferings? Had he not learnt about empathy and duty of care at medical school? Perhaps if he had not skipped the tutorial at medical school on the illness role where the tutor presented Fildes’ painting as the topic for discussion, he may have learnt his lesson much earlier.

## Hypothetical

Consider the following..

The tutor invites the group to look at the painting quietly. The eye goes immediately to the doctor and the young girl because the painter has purposefully placed them not only in the centre but also in full light of the lamp on the table. It is not yet morning. After a minute or two, the tutor asks, *What do you see?* Everyone has an opportunity to speak, several observations are made and after a short while the tutor captures a thread..

Tutor: (to Student 1) *What do you mean about the doctor being ‘intent’?*

Student 1: *What I mean is that the doctor is focused on the young girl.*

Student 2: *I agree. He is intent but then he also looks quite calm, hand on his chin, thinking.*

Student 3: *Maybe thinking.. maybe waiting for something to happen.*

Jack: *For what?*

Student 1: *I’ve read Jane Austen’s ‘Sense and Sensibility’. In those days, when someone had a high fever, from an infection, it was a real worry, I mean, a real worry. Marianne, in the story, who was much older than the girl in this painting, caught a cold from being out in the rain and was very sick. Her mother and her sisters thought that she was going to die.*

Student 2: *Yes, and they waited anxiously for the fever to break, because if it did, which it did in the story, it meant that the patient would live.* (turning to Student 1) *I loved the book!*

Student 3: *But the doctor couldn’t do anything back then. They didn’t have antibiotics like we have now. All they could do was wait.*

Jack: *OK, that’s all the family could do. What’s the point of the doctor sitting there when he couldn’t do anything? And why is Fildes making out that this doctor is some kind of hero when really he isn’t doing anything? He should go home, get some sleep and then, if necessary, come and see the patient in the morning.*

Student 1: *I agree. He can’t do anything. Whether the girl lives or dies has nothing to do with the doctor at that moment but it probably wasn’t practical for him to go home. As happened in ‘Sense and Sensibility’, a doctor often travelled some distance to get to the patient and so it was more practical to stay there..*

Jack: *They could give the doctor a bed at least!*

Tutor: (referring himself to Student 1) *That’s certainly true. It is interesting to speculate about what a doctor could do back then.. and for that matter, what a doctor has the power to do now. But I would like us to shift our focus to the parents, in the shadows. Why has the painter put them in the shadows?*

Student 2: Well, they are quite literally in the dark. They have no idea about their daughter’s illness, whether she will live or die..

Tutor: *What else?*

Student 1: *The mother has her head down on the table, probably crying.*

Student 2: *Exhausted probably. More than likely she’s been up all night.*

Student 3: *Probably crying all night. Run out of tears.*

Tutor: *Look at the father.*

Jack: *He’s just standing there. Staring. He looks useless!*

Student 1: *Be fair, he isn’t just staring. He looks upset.*

Student 2: *Yeah, he’s comforting his wife. He has his hand on her shoulder.*

Jack: *He looks pathetic.*

Tutor: (ignoring Jack’s remark) *So, what’s going on for the parents?*

Student 2: *The mother is distraught. When I had pneumonia last year my mother was beside herself because I was very sick!*

Student 3: *The father doesn’t know what to do. Fathers are always helpless in situations like this. When my mother was in hospital after her car accident, he was looking stunned the whole time. He didn’t know what to say or do.*

Tutor: *So what do you think that they might be feeling, these parents?*

Jack: *I don’t know. I don’t have a daughter to know.*

Tutor: *But what if it was someone really close to you who was seriously sick? How would you feel? You, Student 2, mentioned your mother and you, Student 3, mentioned your father. How do you think they felt?*

Student 2: *I know my mother was really scared. At one point, she thought I was going to die..even though we have antibiotics these days.*

Student 3: *Yeah, my father was scared too. He was really worried, whether my mother would have permanent brain damage, whether she might have to go into a nursing home. Fortunately she came good.*

The tutor is looking at Jack.

Jack: *When you put it that way, if it was someone close to me, say my mother or my father, I guess I would feel helpless.. I would feel as if the whole world was coming to an end.. I would want the doctor to do something.*

Tutor: *But you said earlier that the doctor can’t do anything so he might as well be at home.*

Jack: *I was referring to the doctor in the painting. Then they couldn’t do anything. They didn’t have the medicines and the technology that we have now. Now doctors can do lots of things.*

Tutor: *OK, sure. Let’s just stay focused on what’s going on in this painting, at that time. You said that you would want the doctor to do something.*

Jack: *Well, yes, if there was something that he **could** do then he **should**.*

Tutor: *So, you’re saying, if something changes as regards the girl’s condition..*

Jack: *Yes, that’s right.*

Tutor: *But what if the doctor **had** gone home?*

Jack: *Well he hasn’t. He’s there, waiting.*

Tutor: *But what if it was another doctor who said to the parents, “Not much I can do here. She’s in the hands of God now. I’ll be back in the morning.” What might the parents think about that?*

Student 1: *I think that’s callous and heartless!*

Jack: *If I was the father, I would be very angry and I would insist that the doctor stay.*

Tutor: *Why?*

Jack: *Well, if he, the doctor, returns in the morning it might be too late but if he’s there and something changes, he could do something.*

Tutor: *Is there anything else that the doctor might be doing, other than ‘just waiting’?*

Student 1: *By being there, he is providing support, comfort to the parents.* Student 2: *They’re not alone.*

Tutor: *Indeed. Two parents, helpless, scared that their daughter might die. How lonely might that feel?* The tutor pauses here and after a few moments of silence, adds.. *And what if you were seriously ill, a terminal illness possibly, how would you feel?*

Student 1: *Bloody scared!*

Jack: *It’s not something that I can bear to contemplate. I hope I don’t have to ever face something like that.*

Tutor: *To be ill is to be vulnerable. When you don’t know what will happen to you, it’s very scary.*

Student 3: *Even when you know what’s happening to you! I had gastro a few weeks ago, so bad, I was running to the toilet every 5 minutes, and the stomach pains, so bad, I thought I was going to die. And that was just gastro.*

Tutor: *And that was just gastro.. And so, when you’re sick, you put yourself in the hands of the doctor. Your trust, all your faith and hope is in the doctor..*

## Discussion

Every medical student’s identity is shaped by their experiences at medical school, the medical culture that they come into contact with in clinics and hospitals and of course, by the patients. ( Cruess *et al,* 2015) We cannot know how formative the experience in this hypothetical was for Jack. What we do know is that initially, Jack had fixed views on doctors but gradually, his position changed. A seed was planted and with further experiences in tutorials, interactions with patients and fellow students, and later on fellow doctors, it is quite possible that he will become a more considerate and caring doctor.

In the film, Dr MacKee, having been transformed by his illness experience, makes all his residents spend 72 hours as hospital patients, to learn what it means to be a patient. While we do learn from experience and we can learn many things from our patients, it is not possible to experience everything first hand. It is possible however, to learn and experience many things vicariously through the arts, of various forms. Apart from developing greater awareness and understanding, we also learn to listen more closely to patient narratives. ( Launer, 2012)

Two recent articles reviewed the literature on the use of arts-based initiatives in the medical curriculum. ( Gelgoot, Caufield-Noll and Chisolm, 2018; Taylor, Lehmann and Chisolm, 2018) Both supported the use of the arts and both noted the lack of research with respect to the evaluation of their use and the long-term impact on learners and patients. Such research would certainly be valuable but challenging to conduct because many factors are instrumental in behavioural change. Also, change often takes time and it is difficult to link a specific event to a particular outcome.

The same articles identified various obstacles or difficulties with incorporating the visual arts in medical education. ( Gelgoot, Caufield-Noll and Chisolm, 2018; Taylor, Lehmann and Chisolm, 2018) These included, lack of interest or engagement by learners, because of negative impressions of the arts and, having educators skilled in using the arts. The film *The Doctor* addresses the same themes as Fildes’ painting and they are equally accessible with respect to their themes. Film is perhaps more accessible to modern learners and a medical educator unfamiliar with using the arts would find it relatively easy to use the film to demonstrate its themes and then teach around them. If skill is required, it is in how to elicit and moderate discussion.

The format to follow for discussion is as presented in the hypothetical. Learners should be firstly asked to state what they see and then to expand on the narrative by expressing the thoughts and feelings that are evoked. By guiding, more so than instructing, the educator gradually pulls the pertinent ideas together and focuses the discussion on the intended themes and finally, the intended outcomes. Socratic questioning is a useful way of exploring ideas, guiding discussion in the intended direction, moving the discussion when learners strike difficulty and generally assisting them to develop awareness and understanding. In such a discussion, all beliefs, ideas, thoughts, feelings, have merit. The challenge, for each individual, is to be able to explain themselves and defend their thoughts and beliefs but also to be open to the thoughts and beliefs of others. As with the doctor of the film, meaningful learning occurs as a result of personal discovery. From the richness of the discussion and by being challenged to consider new or alternate possibilities, new meaning is arrived at. This is *transformative learning.* ( Kaufman and Mann, 2007) John Launer would call it *a conversation inviting change.* ( Launer, 2002; Launer, 2012)

What to use from the arts as the medium for instruction does certainly depend on the educator’s familiarity with the arts but also on their ability to be imaginative and creative. Images (painting, drawings, photographs, cartoons) that the educator is familiar with, are a starting point. A visit to the art gallery can be very illuminating. Film is the most accessible of the visual arts and it is not necessary to be a film buff to find a suitable film. There are texts that make specific recommendations about film scenes and the themes to be found. (Alexander, Lenahan and Pavlov, 2015; Alexander, Lenahan and Pavlov, 2012)

There are many articles that describe the arts based initiatives that have been used in various medical schools. ( Gelgoot, Caufield-Noll and Chisolm, 2018; Taylor, Lehmann and Chisolm, 2018) One such initiative is Rita Charon’s school of narrative medicine which has its foundations in literature and uses reflective writing for promoting understanding of patients’ experiences and developing personal insight. ( DasGupta and Charon, 2004; Charon, 2006; Charon, Hermann and Devlin, 2016) The area of creative and reflective writing aside, there is very little on how to use the arts. A good starting point is the ASME publication, ( Gordon and Evans, 2007) from the Understanding Medical Education series, which provides a theoretical framework, discusses what a medical humanities curriculum can offer and how it might be implemented. Powley and Higson’s text ( Powley and Higson, 2005) is a very good resource for the novice with respect to structuring a lesson as well as finding and selecting a resource. Moore’s text (Moore AR, 1978) is a set of transcripts from a medical humanities course which he facilitated and which focused on the non-scientific aspects of being a doctor by discussing excerpts from novels, poems and short stories.

The fact that not all learners are attuned to the arts and might not engage with them is certainly a challenge. It is not unusual for learners to disengage, quite openly sometimes, when they believe that something is superfluous to their learning. Does that mean that educators should address only what the learners consider important and that they should not try to broaden learners’ minds by exposing them to alternate perspectives? In the film *Mona Lisa Smile,* ( Newell, 2003) Katherine Watson, an arts graduate, takes a position as a teacher at a conservative college for young women. Katherine teaches art history but also challenges her students to become independent thinking women, “to see the world through new eyes”. In doing this she meets resistance. In one scene, Katherine takes her students to the storeroom of an art gallery. The students are visibly uncomfortable being in such a place. A Jackson Pollock painting has just been delivered and is unpacked before them. Katherine is in awe, the girls mortified. Katherine’s response to their reaction is simply, “Do me a favour. Do yourselves a favour. Stop talking and look. You’re not required to write a paper, you’re not even required to like it. You **are** required to consider it.” Interestingly, Betty, the student who was most critical of Katherine and her methods, was also the one who in the end was the most appreciative. Because of Katherine’s teaching, Betty was able to address the difficult situations that she was confronted with after leaving school and become an independent woman in her own right. ^[1]^

^[1]^ Both Betty and Dr MacKee undergo personal growth as result of their experiences. The stages of their transformational change are described in *The Hero’s Journey,* a theory developed by Joseph Campbell to explain personal change. The roots of the theory are to be found in myths and legends where the hero undertakes an arduous quest which invariably makes him wiser and stronger in character. Mezirow’s theory of transformative learning has many parallel’s with *The Hero’s Journey.* ( Campbell, 1993; Willans, 2012)

## Concluding remarks

Why should the arts matter when so many astounding scientific and technological advances are occurring daily in medicine? We know so much about disease today and so many diseases are curable like never before. It is perhaps unsurprising that patients and doctors, have come to put their faith in technology, health has become a commodity and the human element has been relegated in importance. ( Tallis, 2004) From the time of Hippocrates there has been tension between patient centredness and disease focus, between the wholistic and reductionist approaches, between precision and uncertainty. We have now moved into the age of artificial intelligence and the artificial doctor and the debate regarding technology versus the human element has been renewed. These tensions will always be there because that is the very nature of medicine. Medicine is both a science and an art. Illness creates uncertainty and a need for certainty. Science and evidence based medicine are valuable tools for treating disease but what happens when the evidence is not there or the disease cannot be cured? What does science have to say about the other side of the illness coin, the human element, the person *with* the disease? The arts generally, not just the visual arts, have from time immemorial grappled with understanding the world around us, what it means to be human, to give substance to what we cannot grasp. Is it not logical to turn to the arts (the humanities) for the lessons which science cannot teach?

## Take Home Messages

• The arts (humanities) should be incorporated into the curriculum because they:
  • teach about the art of medicine
  • broaden awareness and understanding
  • lead to transformational learning and change
• The challenges with respect to the implementation of the arts:
  • are not insurmountable
  • do not require substantial familiarity with the arts either on the educator’s or the learner’s part
• In using the arts, the skill of the educator is in eliciting and moderating discussion around the themes tht are being taught

## Notes On Contributors

Dr George Zaharias is a General Practitioner and Medical Educator, in Melbourne, Australia. His field of expertise is remediation, having managed the remediation of Registrars in a General Practice training program for over 10 years. In his current role with the Royal Australian College of General Practitioners, his responsibilities relate to the remediation of General Practice Registrars and General Practitioners as well as assisting General Practitioners with returning to work.
