# Supplementary material for: A visit to The Doctor
Source: MedEdPublish (2016). 2018 Sep 4;7:191. [Version 1] doi: 10.15694/mep.2018.0000191.1 (PMC10701857; doi:10.15694/mep.2018.0000191.1)
Supplement: Peer review history

# Reviewer report: Reviewer response for version 1

**DOI:** 10.21956/mep.19656.r29323

**Published:** 2018-09-18

**Authors:** Jonathan McFarland

**Affiliations:** 1 Sechenov University, Moscow

This review has been migrated. The reviewer awarded 4 stars out of 5 I found this a fascinating article and also a very good read. Both interesting and fun, which is a word not often used in scientific literature but perhaps one that should be sought more often. At the end of the article, the author asks the question, "Is it not logical to turn to the arts ( the humanities) for the lessons which science cannot teach?", and I truly believe that this question should be printed out in bold and placed on the desks of all policymakers involved in medical education, as it is so unequivocally correct. Both works are known to me, and what I enjoyed greatly, and found very stimulating and pertinent, was the author's interlinking of Fildes' painting and Haines' film through the Hypothetical section where the tutor invites his group of students to study the painting. Great imaginative writing but with an important message, and in this article, I think that we could finally be beginning to get to grips with "How" to introduce the arts into medical education. All in all, this should be read by all those with an interest in this topic.

Reviewer Expertise:

NA

No decision status is available

# Reviewer report: Reviewer response for version 1

**DOI:** 10.21956/mep.19656.r29322

**Published:** 2018-09-05

**Authors:** Tracey Collett

**Affiliations:** 1 Plymouth University Peninsula Medical School

This review has been migrated. The reviewer awarded 4 stars out of 5 I really enjoyed reading this paper. The title captured my imagination and the paper is a good read. I think that the arts are really good for teaching awareness of the art of medicine and also some aspects of sociology and psychology in medicine. I particularly enjoyed how Dr Zaharias used the dialogue to demonstrate socratic questioning. Attention is frequently paid to how our perspectives can be transformed by others as we come across them in everyday life (for example a stranger, a doctor who demonstrated humanity, a friend sharing experiences of working in complex settings) however often as lecturers we present information factually via powerpoint. This paper suggests that education should be a process of dialogue in which educatees and educators engaged in mutually respectful learning. I think it is useful for teachers and have printed it off as a resource.

Reviewer Expertise:

NA

No decision status is available

# Reviewer report: Reviewer response for version 1

**DOI:** 10.21956/mep.19656.r29324

**Published:** 2018-09-04

**Authors:** Trevor Gibbs

**Affiliations:** 1 AMEE

This review has been migrated. The reviewer awarded 4 stars out of 5 A very interesting paper and one very neatly written in my view, fully supporting the value of using the arts, together with film and books to bring value to our teaching and to create more transformative learning- getting the students to reflect, to think differently, to internalise and then express their feelings and to place themselves in the context of the patient through these observations. I really enjoyed reading this paper and its content gives definition to what the Humanities can bring to medical education and the development of our future Doctors.As someone who uses this painting in my own teaching, I would disagree somewhat with the authors second bullet point in the second Take Home Message. I do think that knowing something about the background can add value. In this case and knowing that different theories exist as to the painting's origin , the painting is most likely based upon Fildes' own experience of the death of his son, and how he saw the compassion and worthlessness within the doctor. I often ask the students to reflect on that; how would they feel if they had no cure for a patient who would die from what they perceive is a simple illness?Anyone interested in the Humanities or involved in teaching the Humanities should read this paper

Reviewer Expertise:

NA

No decision status is available

# Reviewer report: Reviewer response for version 1

**DOI:** 10.21956/mep.19656.r29325

**Published:** 2018-09-04

**Authors:** P Ravi Shankar

**Affiliations:** 1 American International Medical University

This review has been migrated. The reviewer awarded 4 stars out of 5 This is an interesting manuscript using two specific examples to discuss about the use of the arts in medical humanities education. I and my colleagues generally use the painting ‘The doctor’ in all of our medical humanities (MH) modules. One of the points to consider as mentioned by the author was why the doctor who could do so little (the one depicted in the painting) was more respected and accepted than the modern day doctor with a vast therapeutic arsenal. We had also used the movie in our session on movie screening and activities which we had conducted at Aruba. The manuscript is well written but can be strengthened by also including initiatives from the developing world. Slowly but surely the developing nations are becoming more active in the humanities. There are various programs utilizing art in the education of doctors in developing nations. In India, the Centre for community dialogue and change (CCDC) in Bengaluru conducts workshops on the theater of the oppressed (TO). Having attended a workshop myself, I am sure there are various benefits from using TO in health professions education. The author also describes the challenges involved in measuring the impact of the humanities. The manuscript will be of interest to all medical educators with a special interest in the medical humanities.

Reviewer Expertise:

NA

No decision status is available
